# Comparison of microscopic full-laminectomy (open surgery) and microendoscopic minimally invasive hemilaminectomy for thoracic extramedullary spinal tumours

**DOI:** 10.1186/s13019-024-02969-4

**Published:** 2024-07-13

**Authors:** Gang Chen, Yong Yu, Chengxing Qian, Yong Jiang, Jie Chen

**Affiliations:** Department of Neurosurgery, Tongling People’s Hospital, No.468, Tongling, 244099 Anhui People’s Republic of China

**Keywords:** Endoscopic surgery, Tubular retractor, Thoracic spinal tumours, Minimally invasive

## Abstract

**Background:**

Minimally invasive treatments for spinal cord tumours are common. The aim of this study was to compare the perioperative outcomes of patients with thoracic extramedullary spinal tumours (TEST) treated by microendoscopic minimally invasive surgery—hemilaminectomy through a homemade tubular retractor (MIS-TR) and microscopic full laminectomy (open surgery).

**Methods:**

Between February 2016 and February 2021, 51 patients with TEST were included. According to their clinical data, patients were classified into the MIS-TR group (*n* = 30) and the open surgery group (*n* = 21) and assessed.

**Results:**

In both groups, the mean operation time, change in perioperative ASIA score, and modified Macnab score were comparable. The average postoperative hospital stay in the MIS-TR group was substantially shorter than that in the open surgery group (*p* < 0.0001). The mean blood loss volume in the MIS-TR group was substantially lower than that in the open surgery group (*p* = 0.001). The perioperative complication rate in the MIS-TR group was considerably lower than that in the open surgery group (*p* < 0.0001). At the 3-month follow-up, there was no substantial difference in the Oswestry Disability Index (ODI) score improvement between the two groups. Nonetheless, at the 12-month follow-up, the average ODI in the MIS-TR group was considerably lower than that in the open surgery group (*p* = 0.023). The main influencing factors for complete postoperative recovery were preoperative ASIA score (OR 7.848, *P* = 0.002), surgical complications (OR 0.017, *P* = 0.008) and age (OR 0.974, *P* = 0.393).

**Conclusions:**

MIS-TR is safer and more effective than open surgery for treating TEST, but the long-term recovery of MIS-TR is not better than that of open surgery.

**Supplementary Information:**

The online version contains supplementary material available at 10.1186/s13019-024-02969-4.

## Introduction

Because of the rigid construction of the spinal canal and neural foramina, spinal cord tumours, albeit often of benign histopathologic origin, can cause compressive myelopathy and radiculopathy [1]. Gross-total resection (GTR) with minimal neurological deficit is the principle of surgery for maintaining spinal stability. Due to substantial soft-tissue dissection and disruption of midline structures involved with open surgical techniques, a minimally invasive surgery (MIS) approach involving tubular retractors (TRs) has become more popular for treating spinal pathologies [2, 3]. TR and microsurgical approaches are safe and effective for use in minimally invasive intracerebral haematoma evacuation, [4] spinal tumour excision (intradural and extradural), [5] and extraforaminal L5/S1 microdiskectomy [6]. 

Endoscopic neurosurgical techniques offer superior visualization of deep lesions with less retraction and invasion of important functional structures [7–9]. MIS had a considerably lower predicted blood loss volume, shorter surgical time, and shorter length of stay while remaining safe and maintaining high rates of gross-total resection [10]. Laminectomy has traditionally been the favoured surgical method for resecting spinal tumours. Recent trends in the efficacy hemilaminectomy as a minimally invasive and viable alternative surgical method has sparked interest in its use for resecting spine malignancies [11]. When deciding on a method, tumour histology and location are critical factors to consider. A large series of spinal tumours treated with laminectomy and hemilaminectomy revealed that benign juxtamedullary tumours were excellent candidates for hemilaminectomies, but malignant tumours with complicated morphology require bilateral laminectomy for optimal exposure and resection [12]. 

The adoption of minimally invasive treatments that expedite postoperative recovery and lower the risk of complications and systemic surgical stress may result in improved cancer survival and certainly has a role in accelerating patients’ return home and continuation of oncologic therapy [10, 13]. However, other important factors to consider when selecting a surgical corridor for a spinal tumour are the maintenance of spinal stability, [11] and sufficient thoracic canal space [14]. 

At our institute, we explored a MIS-hemilaminectomy technique employing a homemade tubular retractor (MIS-TR) vs. a routine open operative approach for resecting thoracic extramedullary spinal tumours (TEST).

## Materials and methods

### Patients

This was a retrospective study involving a total of 51 TEST patients, all of whom underwent tumour resection in the Department of Neurosurgery at our hospital between February 2016 and February 2021. The Ethics Committee of Tongling People’s Hospital approved this study (No. 202,216). Thirty patients underwent minimally invasive tubular retractor (MIS-TR group) resection of the TEST tumours according to their willingness after the different surgical methods were explained. We compared the outcomes of patients with those of 21 patients who underwent routine procedures (microendoscopic hemilaminectomies, open surgery group). The sagittal and axial diameters of the tumours ranged from 0.5 ∼ 3.2 cm and 1.2 ∼ 2.9 cm, respectively. Inclusion criteria: ①All patients who underwent surgery for intraspinal tumours were included for the first time. ② the TEST extended up to two centrums. ③there was no spine instability or spinal structure destruction. ④ complete clinical and pathological data. ⑤patients who were followed up as outpatients or via telephone follow-up for at least 2 years. Patients with vascular tumours, tumour recurrence, spinal destruction or instability were excluded.

### Surgical technique

Routine open surgery was performed. The procedure was performed through a posterior median approach with complete resection of the spinous process and laminectomy and microscopic removal of the tumour.

**Fig. 1:**
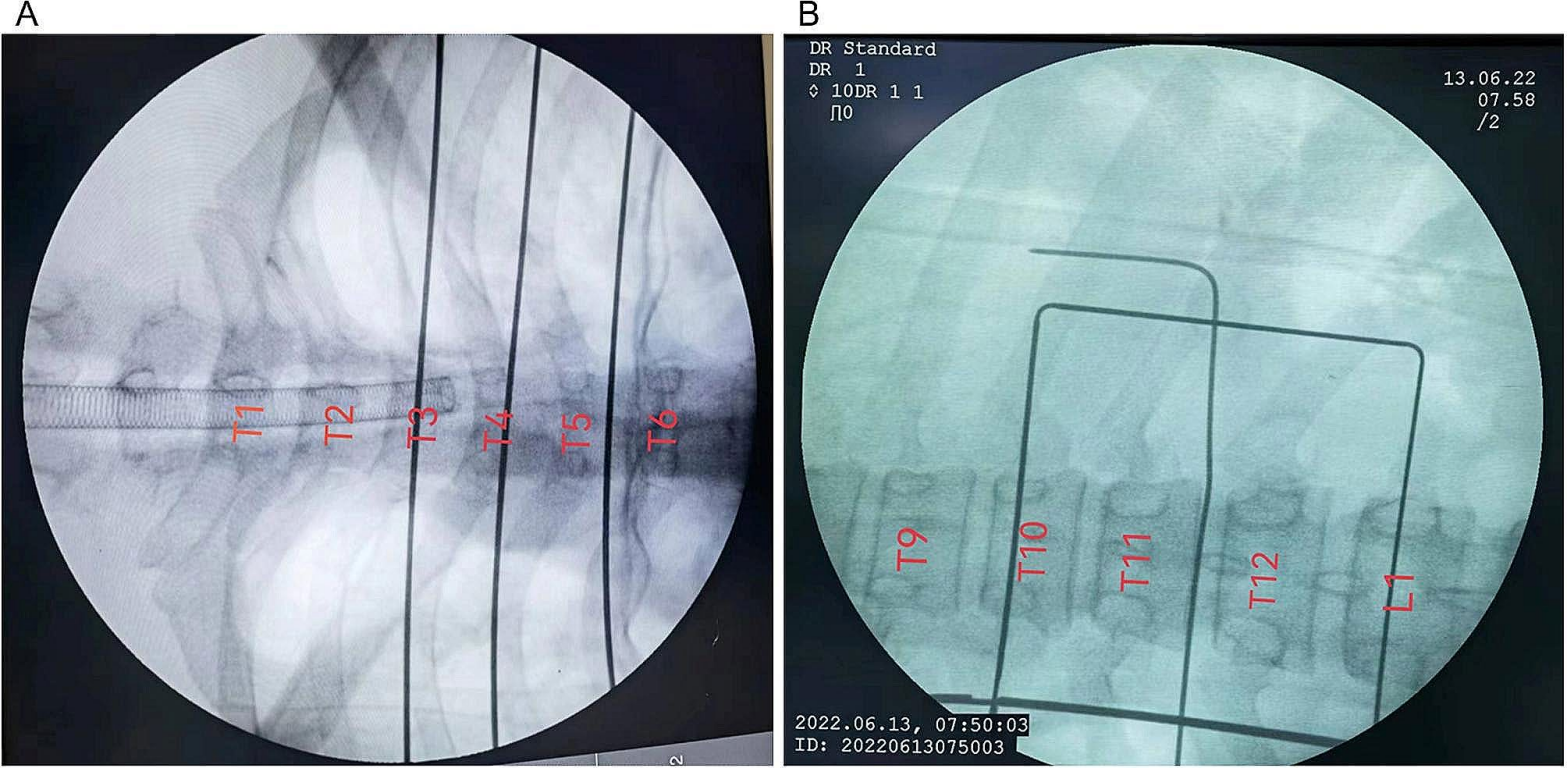
**A** and **B**: Using metal needles, the T1–T12 thoracic vertebral body segments were marked with a C-arm. Each X-ray image had two neighbouring vertebral body metal markers for tumour localization

MIS-TR patients were positioned prone following general anaesthesia. The head was fixed to allow treatment for upper thoracic lesions. The level of the tumour was identified using a C-arm before the skin was incised. The operator first marked the T1-T12 thoracic vertebral body segments in vitro with a MARK pen and secured the kerf pins with adhesive tape (Fig. 1A). Then, the C-arm was moved from T1 to T12 to obtain X-ray images of the number of marked vertebral bodies where the tumour was located, with each X-ray image containing two adjacent vertebral body-metal markers for tumour location (Fig. 1B). The retraction of the spinal cord and neurological function were detected by intraoperative neurophysiological monitoring of somatosensory and motor evoked potentials.

The lamina was exposed lateral to the facet joints via dissection after a midline incision across the spinous processes. The dura was fully exposed, and the bilateral lamina and ligaments were removed both above and below the tumour boundary. The tumour was resected via standard microsurgical techniques under an operating microscope. Prolene 5 − 0 sutures were used for watertight closure of the dura. Interrupted absorbable sutures were used to close the fascia and subcutaneous tissue.

**Fig. 2:**
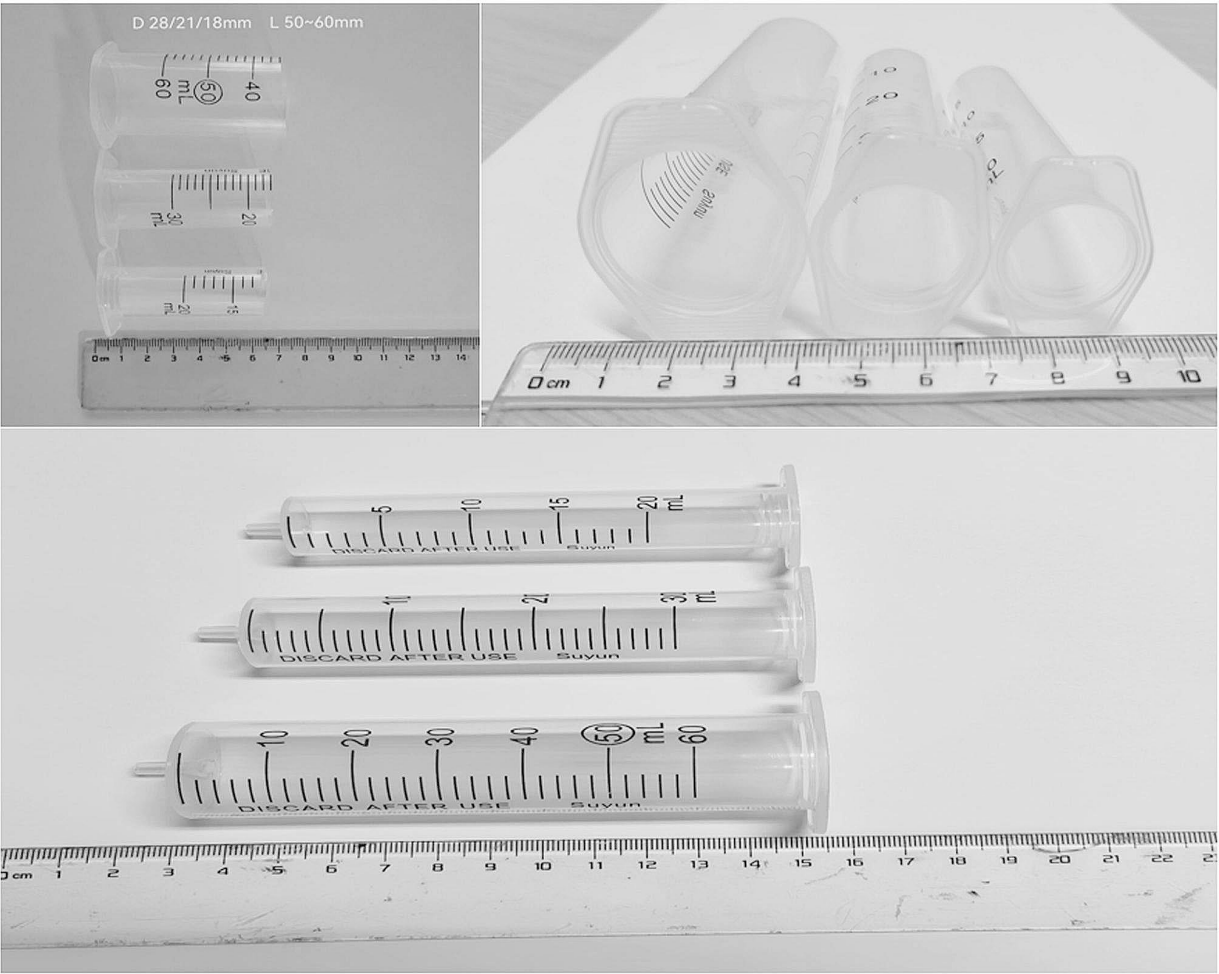
Homemade tubular retractor

**Fig. 3:**
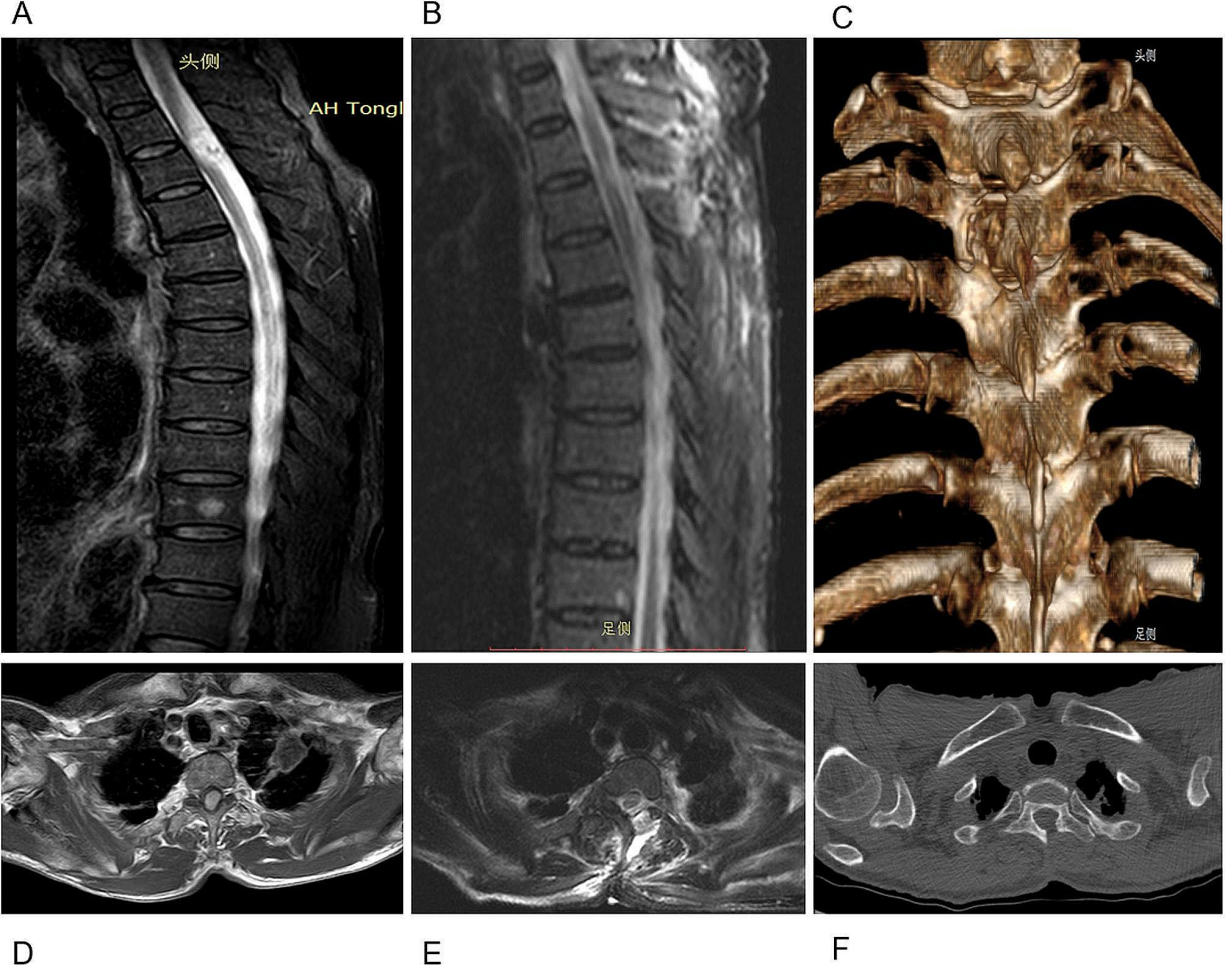
Endoscopic surgery through a tubular retractor for thoracic intradural schwannomas. **A** and **D**: Preoperative sagittal T2-weighted (**A**) and axial T1-weighted contrast-enhanced (**D**) MR images suggest a T2 intradural-extramedullary schwannoma. **B, C, E** and **F**: Postoperative sagittal (**B**) and axial (**E**) T2-weighted MR images demonstrating GTR with limited left hemilaminectomy, as shown by postoperative 3D CT reconstruction (**C**) and axial CT (**F**)

**Fig. 4:**
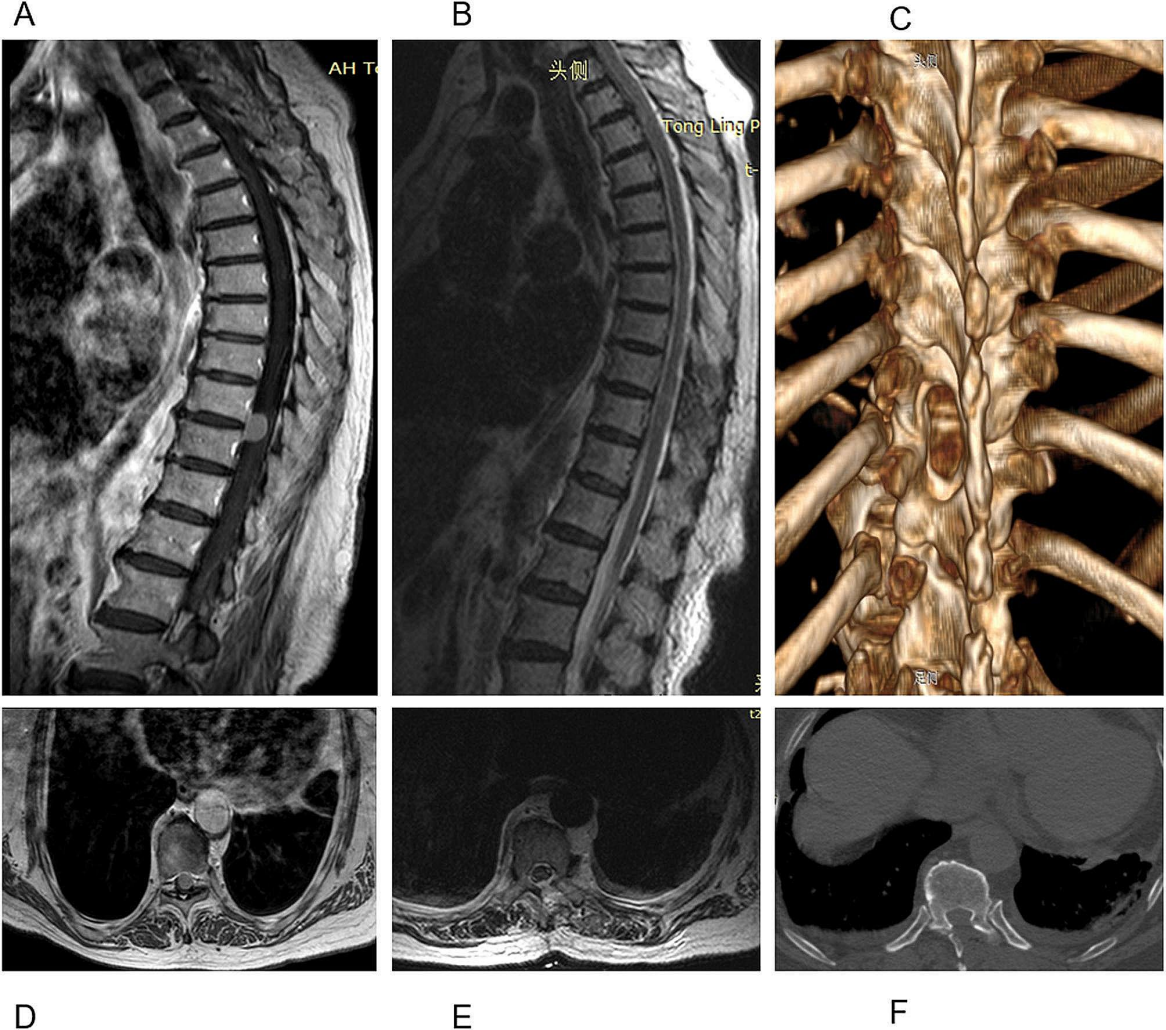
Endoscopic surgery through a tubular retractor for thoracic intradural meningioma. **A** and **D**: Preoperative sagittal (**A**) and axial (**D**) T1-weighted contrast-enhanced MR images suggest a T10-11 intradural-extramedullary meningioma. **B, C, E** and **F**: Postoperative sagittal (**B**) and axial (**E**) T2-weighted MR images demonstrating GTR with limited left hemilaminectomy, as shown by postoperative 3D CT reconstruction (**C**) and axial CT (**F**)

**Fig. 5:**
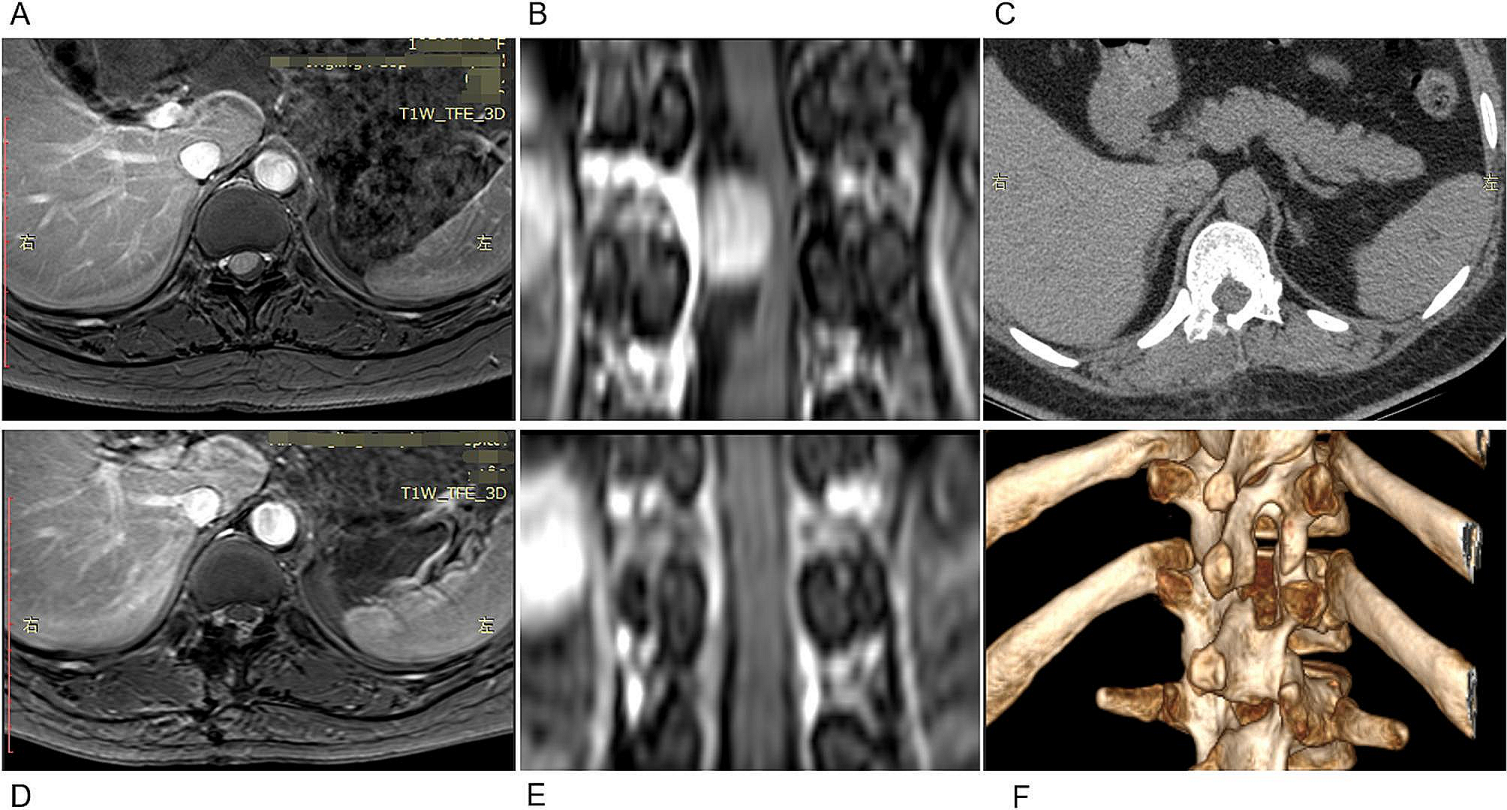
Endoscopic surgery through a tubular retractor for thoracic intradural schwannomas. **A** and **B**: Preoperative axial (**A**) and coronal (**B**) T1-weighted contrast-enhanced MR images suggest a T12 intradural-extramedullary schwannoma. **C, D, E** and **F**: Postoperative axial (**D**) and coronal (**E**) T1-weighted contrast-enhanced MR images demonstrating GTR with limited right hemilaminectomy, as shown on the postoperative axial CT scan (**C**) and 3D CT reconstruction (**F**)

MIS started with a 2.5 ∼ 4.0 cm skin incision 1.0 ∼ 2.0 cm lateral to the midline and localized over the centre of the tumour. Surgical access was achieved utilizing dilation via stout forceps and finger dissection. Then, a 20-mm non-expansile homemade tubular retractor (Fig. 2) was fixed in place towards the desired tumour level with a table-mounted flexible arm (the tubular retractor was fastened on the ipsilateral side of the operator with a snakeskin-like stent, and the endoscopic stent arm supported and secured the endoscope on the contralateral side). Next, a 4-mm-diameter, 18-cm-long 0-degree neurosurgical endoscope was inserted. The medial facet portion, if necessary, and the ipsilateral lamina were excised. Using a drill, the base of the spinous process and the lamina on the opposing side (hemi-laminectomy) were undercut [15]. To retract the dural window and fully expose the subdural area, 6 − 0 Prolene sutures were used to suspend the sliced dura over the soft tissue close to the bone window. Microsurgical expertise and a bimanual method were used to remove the tumour. An endoscope holder may be employed to enable the surgeon to perform the surgery with both hands free. The sliding knot method was used to ensure watertight closure of the dura, at which time the 6 − 0 Prolene sutures were cut short so that they could be manipulated under endoscopic view [16]. The gradual withdrawal of the tube allowed for strict haemostasis in every dissection plane, with no dead space between them (simulated situations are illustrated in Figs. 3A-F and 4A-F). A comparison of the preoperative and postoperative conditions of one patient is shown in Fig. 5A-F.

### Clinical evaluation

All patients were monitored for a minimum of one year (12 months). Data such as age, sex, tumour location, pathology, operating time, blood loss volume, length of hospital stay, postoperative modified MacNab scores, [17, 18] and complications were summarized, computed, and compared (Table 1). Aside from a physical examination, each patient underwent regular radiography with dynamic imaging and spinal magnetic resonance imaging to rule out any tumour recurrence and to detect any spinal instability or deformity. An impartial surgeon assessed the neurologic state using the American Spinal Injury Association Impairment Scale (ASIA classification) [19]. Patients’ functional statuses were examined using the Oswestry Disability Index (ODI) [20] before surgery, three months after surgery, and twelve months after surgery.

**Table 1:** Comparison of patient data between the two groups

Groups	MIS(30)	Open(21)	*P* value
Male/Female	13/17	8/13	0.708
Age(years)	56.5 ± 14.9	58.4 ± 13.8	0.655
Symptoms			0.880
Back pain	22	12	
Radicular symptoms	16	13	
Motor deficits	19	14	
Bladder dysfunction	4	3	
Tumour level			1.000
Upper T1-T6	10	7	
Lower T7-T12	20	14	
Location			0.912
Extradural	4	2	
Intradural	23	17	
Dumbbell	3	2	
Pathology			0.888
Neurinoma	17	11	
Meningioma	11	9	
Others	2	1	
Neurofibroma	1	1	
Cytoglioma	1		
Operation			
Operative time(min)	185.3 ± 48.5	205.7 ± 62.4	0.196
Blood loss(ml)	118.7 ± 72.7	211.9 ± 116.1	0.001
Postoperative stay(days)	7.6 ± 1.5	10.5 ± 2.0	0.000
Complications (%)	1 (3.3%)	5 (23.8%)	0.000
CSF leak	0	2	0.085
Intracranial infection	0	1	0.227
Vesicorectal disorder	1	1	0.796
Epidural hydroma	0	1	0.227
ASIA score (postoperative vs. preoperative)			0.182
better	27	16	
same	3	5	
worse	0	0	
ODI scores(%)			
Before operation	65.7 ± 19.6	66.1 ± 16.8	0.935
3-months follow up	31.2 ± 19.4	38.3 ± 20.9	0.220
12-months follow up	10.0 ± 11.4	18.8 ± 15.1	0.023
Modified MacNab score(12-months)			0.352
Excellent	13	8	
Good	15	8	
Fair	1	4	
Poor	1	1	

CSF cerebrospinal fluid, ASIA American Spinal Injury Association Impairment Scale, ODI Oswestry Disability Index

### Statistical analysis

The measurement data were recorded as the mean ± standard deviation in IBM SPSS 25.0 statistical software (Armonk, NY, USA) for the statistical analysis. Independent samples t tests, chi-square tests, Fisher’s exact tests, and Mann-Whitney U tests were used for data analysis. A *p* value less than 0.05 was regarded as statistically significant. The influence of each component on overall rehabilitation was assessed using univariate and multivariate logistic regression analyses (defined as a 12-month postoperative ASIA grade E). The power of the model was predicted using the receiver operating characteristic (ROC) curve.

## Results

### Comparison of generic information between the two groups

The mean age of the patients in the MIS-TR group was similar to that of the patients in the open surgery group (56.5 years to 58.4 years, Table 1). The sex distributions of the two groups were comparable (*P* = 0.708). Considering the symptoms, tumour level, tumour location, pathology, and operation time, the two groups were comparable (*P* > 0.05, Table 1). The sagittal and axial diameters of the tumours ranged from 0.5 ∼ 3.2 cm and 1.2 ∼ 2.9 cm, respectively (*P* > 0.05). There were no conversions to open surgery.

The average postoperative hospital stay in the MIS-TR group was substantially shorter than that in the open surgery group (7.6 days versus 10.5 days, *p* < 0.0001; Table 1). The mean blood loss volume in the MIS-TR group (118.7 ml) was considerably lower than that in the open surgery group (211.9 ml) (*p* = 0.001, Table 1).

### Incidence of perioperative complications

The perioperative complication rate in the MIS-TR group was considerably lower than that in the open surgery group. One perioperative complication occurred in the MIS-TR group (3.3%), and five complications occurred in the open surgery group (23.8%) (*p* < 0.0001, Table 1). In the MIS-TR group, only one patient had vesicorectal disease. The complications in the open surgery group included two cases of cerebrospinal fluid (CSF) leakage, one instance of cerebral infection, one case of vesicorectal dysfunction, and one case of epidural hydroma (Table 1). There were no deaths in our series.

### Evaluating neurological conditions before and after surgery

The ASIA classification and ODI score were used to assess neurological state before surgery, three months after surgery, and twelve months after surgery. At the 12-month follow-up, the improvement from the preoperative ASIA score to the 12-month postoperative ASIA score was similar across the two groups: 76.7% better, 23.3% same in the MIS-TR group and 66.7% better, 33.3% same in the open surgery group, as illustrated in Tables 1 and 2. In both groups, postoperative ODI scores decreased with time and considerably improved compared to those before surgery. At the 3-month follow-up, there was no discernible improvement in the ODI score between the two groups (*p* = 0.220, Table 1), but at the 12-month follow-up, there was a discernible difference (*p* = 0.023, Table 1). At the 12-month follow-up, the modified MacNab scores in both groups were similar (*p* = 0.352).

**Table 2:** Neurological status evaluated by the ASIA classification system

ASIA grades	A	B	C	D	E	*P* values
Before operation						0.525
open surgery group	0	2	7	10	2	
MIS-TR group	0	3	7	16	4	
3-months follow-up						0.828
open surgery group	0	2	0	13	6	
MIS-TR group	0	3	5	11	11	
12-months follow-up						0.848
open surgery group	0	1	0	5	15	
MIS-TR group	0	1	2	5	21	

### Predictive modelling for full rehabilitation

**Fig. 6:**
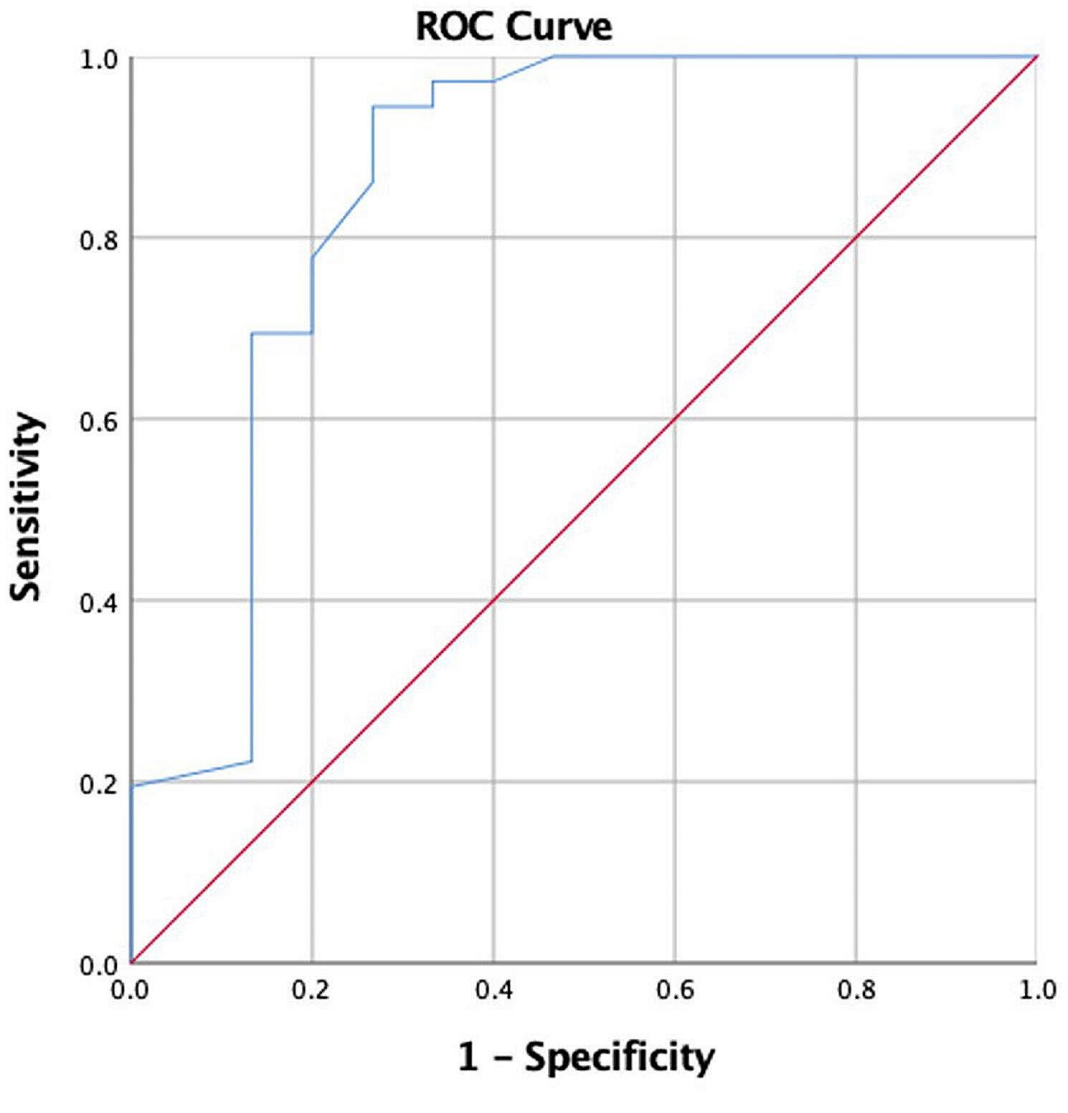
Receiver operating characteristic (ROC) curve for our prognostic model for complete rehabilitation (defined as a 12-month postoperative ASIA grade E) showing the area under the curve (AUC = 0.856)

Age, sex, tumour location, complete ventral location, tumour level (upper T1-T6/lower T7-T12), pathology, preoperative ASIA score, surgical approach (open or MIS), and complications (yes or no) were found to be significantly associated with complete rehabilitation (defined as a 12-month postoperative ASIA grade E) (Table 3). The ASIA score (*p* = 0.003), comorbidities (*p* = 0.071), and age (*p* = 0.045) were all strongly related to a full recovery. Next, we conducted multivariate logistic analysis using all significant variables (*p* < 0.1) from the univariate model (Table 4). The ROC curve indicated that this model has reasonable predictive power (area under the curve = 0.856) (Fig. 6).

**Table 3:** Univariate analysis of the predictors for complete rehabilitation (defined as 12-month postoperative ASIA grade E)

Variables	Odds ratio	*P* values
Age	0.952	0.045
Sex	1.071	0.912
Pathology		0.384
Meningioma/schwannoma	0.545	0.643
Other/schwannoma	0.750	0.826
Location		0.688
Extradural/intradural	0.000	0.999
Dumbbell/intradural	0.000	0.999
Levels (lower T7-T12/upper T1-T6)	2.625	0.135
Complete ventral location	0.382	0.361
Surgical approach (MIS/open)	1.071	0.912
Complications	0.114	0.071
Preoperative ASIA grade	4.226	0.003

**Table 4:** Multivariate analysis of the predictors for complete rehabilitation (defined as 12-month postoperative ASIA grade E)

Variables	Odds ratio	Standard error	*P* values	95% CI
Preoperative ASIA grade	7.848	0.664	0.002	2.137–28.821
Complications	0.017	1.530	0.008	0.001–0.345
Age	0.974	0.031	0.393	0.916–1.035

## Discussion

The aim of gold-standard treatment for spinal tumours is complete tumour removal with minimal neurological deficit while maintaining spinal stability [21]. Thoracic spinal tumours are generally regarded as challenging for surgeons because of their kyphotic curvature, narrow canal, high spinal cord-to-canal space ratio, and limited blood supply [22]. 

The contralateral paraspinal muscles and the posterior spinal components are destroyed with the typical open technique, which increases the risk of postoperative problems and spinal instability [23]. MIS was developed to preserve the structural and functional integrity of the spine and to reduce the incidence of approach-related destabilization. MIS has been proven to be effective and safe for most extramedullary neoplasms [5, 7, 8, 24–26]. MIS-TR was proven to be effective in treating spinal tumours by Balasubramanian et al. [5]. Endoscopic MIS for intraspinal malignancies has been proven to be safe and successful with a panoramic view and close-up observation [15, 27]. For a ventral tumour, facet joint excision might be reduced or avoided [28]. Previous research has mostly concentrated on microsurgery or extramedullary malignancies in all spinal segments [29]. 

The perioperative outcomes of patients with thoracic spinal tumours who underwent resection via MIS-TR or open surgery were examined in this research. Age, sex distribution, preoperative symptoms, and tumour site were comparable between the MIS-TR group and the open surgery group. There was no substantial difference in pathological findings, preoperative ASIA score, preoperative ODI score or mean operative time. This study revealed significant differences in operative blood loss volume and postoperative length of stay between the two groups, similar to previous studies [5]. The average postoperative length of hospital stay in the open surgery group was substantially longer than that in the MIS-TR group. This difference may be due to the unilateral muscle-splitting approach, smaller soft-tissue dissection, lower postoperative pain, and no CSF leakage in the MIS-TR group. Compared with open surgery, MIS-TR had a significantly lower complication rate. These results are comparable to the previous literature [12, 30]. 

In our investigation, the MIS-TR group had no CSF leakage, while the leakage rate was 9.5% in the open surgery group. Because of minimal soft-tissue exposure, the smaller surgical cavity, and dead space, the tubular technique reduces the likelihood of postoperative symptomatic CSF leakage. The perioperative ASIA score, modified MacNab score, and 3-month follow-up ODI score did not significantly differ between the two groups. This demonstrates that thoracic spinal tumours may be safely and efficiently treated using either open surgery or MIS.

The 12-month follow-up ODI score differed significantly between the two groups. The reason for this might be that the ODI score differs from the ASIA grade. Compared to the ASIA classification, the ODI score examined pain and spinal motor function in addition to neural condition. This might be because the ligamentum, muscle, and flexion motion are better maintained in MIS than in open surgery, which reduces postoperative discomfort and improves postoperative spinal motor function. This finding implies that MIS is more suitable for long-term functional rehabilitation than is open surgery.

This study revealed that the preoperative ASIA grade, incidence of complications, and age were strongly related to neurological recovery, which is consistent with prior research [15, 31–33]. Neither the tumour level nor the whole degree of ventral placement were associated with neurological recovery. This differs from what Mehta [34] reported. Patients with upper thoracic spinal tumours, particularly full ventral tumours, most likely had postoperative neurological impairments. According to earlier research, neurological recovery differs greatly depending on the severity of the SCI, in the following order: C > B > D > A [35]. In the research by Skeers et al., patients with Grade A showed more compression than did those with incomplete motor damage, [36] and ASIA grade A was linked to a higher risk of severe neurological impairments. Additionally, according to Kirshblum et al., [37] patients with incomplete sensory tetraplegia (ASIA grade B) recover significantly more sensory function than do patients with initial ASIA grade A, which also suggests that the neurological recovery of patients with an initial ASIA grade A was less successful than that of patients with an initial ASIA grade B. This finding may be the result of the sample size or the extra care we take with these tumours to prevent consequences.

Although endoscopic surgery using tubular retractors has been demonstrated to be a promising and beneficial treatment in previous publications, including this investigation, tumour debulking is suggested to prevent the manipulation of nerve structures, and intraoperative neuromonitoring is essential [38]. Tumours involving two or more layers, haemorrhagic tumours, tumours spreading to both sides, and intramedullary neoplasms are all relative contraindications to this method. Furthermore, because of the limited room for executing typical procedures, hermetic dural closure is a significant issue. Small needle size, hand rotation rather than linear movement [15], extracorporeal sliding knots, [16] and the use of a U-clip [39] might be beneficial for this purpose. To improve dural repair, fibrin glue might be used [15]. 

This study suggested that MIS is more conducive to short-term and long-term recovery than is open surgery. Preoperative ASIA scores, complications, and age are strong predictors of complete postoperative recovery. However, these findings need to be elucidated by more extensive randomized, prospective trials. However, this study has several limitations. First, the sample size was small, and the study was retrospective, which could lead to bias. Second, the follow-up period was 12 months, and a longer period of evaluation is needed in future studies to obtain more clinically relevant data.

## Conclusion

In conclusion, the results of this study indicate that TEST can be safely and effectively treated endoscopically with a tubular retractor. Treatment of thoracic spine tumours with MIS resulted in significantly less surgical blood loss, shorter postoperative stays, fewer complications, and better ODI scores at the 12-month follow-up than did open surgery.

### Electronic supplementary material

Below is the link to the electronic supplementary material.

Supplementary Material 1

## Data Availability

No datasets were generated or analysed during the current study.
