# Supplementary material for: Comparison of microscopic full-laminectomy (open surgery) and microendoscopic minimally invasive hemilaminectomy for thoracic extramedullary spinal tumours
Source: J Cardiothorac Surg. 2024 Jul 13;19:444. doi: 10.1186/s13019-024-02969-4 (PMC11245853; doi:10.1186/s13019-024-02969-4)
Supplement: Supplementary file 1 — Supplementary Material 1 [file 13019_2024_2969_MOESM1_ESM.pdf]

SPRINGER NATURE
Author Services

Editing Certificate

This document certifies that the manuscript

Comparison of microscopic full-laminectomy (open surgery) and microendoscopic minimally invasive surgery - hemi-laminectomy for thoracic extramedullary spinal tumors

prepared by the authors

Gang Chen, Yong Yu, Chengxing Qian, Yong Jiang, Jie Chen

was edited for proper English language, grammar, punctuation, spelling, and overall style by one or more of the highly qualified native English speaking editors at SNAS.

This certificate was issued on **May 31, 2024** and may be verified on the SNAS website using the verification code **2FEA-9CAD-AC43-4F7D-0C43**.

Neither the research content nor the authors' intentions were altered in any way during the editing process. Documents receiving this certification should be English-ready for publication; however, the author has the ability to accept or reject our suggestions and changes. To verify the final SNAS edited version, please visit our verification page at secure.authorservices.springernature.com/certificate/verify.
If you have any questions or concerns about this edited document, please contact SNAS at support@as.springernature.com.

SNAS provides a range of editing, translation, and manuscript services for researchers and publishers around the world.
For more information about our company, services, and partner discounts, please visit authorservices.springernature.com.
